# The Shape Trail Test Is Sensitive in Differentiating Older Adults with Mild Cognitive Impairment: A Culture-neutral Five-minute Test

**DOI:** 10.14283/jpad.2024.80

**Published:** 2024-05-02

**Authors:** Z. Ding, Agnes S. Chan

**Affiliations:** 1 Department of Psychology, The Chinese University of Hong Kong, Room 355, Sino Building, New Territories, Hong Kong SAR, Shatin, China; 2 Research Centre for Neuropsychological Well-being, The Chinese University of Hong Kong, Shatin, New Territories, Hong Kong SAR, China

**Keywords:** Shape Trail Test, Trail Making Test, mild cognitive impairment, subjective memory impairment, diagnostic accuracy

## Abstract

**Introduction:**

The Shape Trail Test (STT) was developed based upon the Trail Making Test, as a culture-neutral test for measuring processing speed and mental flexibility. This study aims to evaluate the accuracy and validity of this five-minute test for differentiating individuals with normal cognition (NC), subjective memory impairment (SMI), and mild cognitive impairment (MCI).

**Method:**

The study included 210 participants aged 50–80 years, with 70 participants in each group matched for age, education, and gender.

**Results:**

No significant difference in STT measures was found between the NC and SMI groups. In contrast, both the NC and SMI groups exhibited significantly better performance (shorter completion time in STT-A and STT-B and fewer STT-B errors) than the MCI group. No significant group differences were found in STT-A errors. Stepwise regression analysis identified three significant predictors for classifying the MCI group from the NC and/or SMI groups, including the STT-B completion time, the STT-A errors, and the interaction between STT-B completion time and STT-B errors. The composite score of these three predictors demonstrated good discriminatory power for classifying the MCI group from the other groups, with area under the curves (AUCs) of 0.76–0.79 (p < 0.001), sensitivities of 78.6%–80%, and specificities of 60%–61.4%. However, none of the STT measures or their interactions were significant predictors for differentiating the SMI group from the NC group. Besides, the STT measures were significantly correlated with age, education, and executive function measures.

**Discussion:**

The STT could be a culture- and language-free, reliable test for assessing executive function and a sensitive test for predicting MCI.

**Electronic Supplementary Material:**

Supplementary material is available in the online version of this article at 10.14283/jpad.2024.80.

## Introduction

Shape Trail Test (STT) is a culture-neutral test for assessing processing speed, mental flexibility, attention, sequence, and visuomotor skills. It was developed by Agnes Chan and her research team in 1997 and has been widely used in the Chinese population since then. It was developed based on the Trail Making Test (TMT), which relies on Latin alphabets and is less applicable to populations using non-alphabetic languages. To overcome this limitation, the STT requires participants to connect numbers 1–25 in order, alternating between two different enclosing shapes.

Our research team has applied the STT in diverse populations. An early study used the STT to evaluate the effect of an herbal remedy on executive functions in Chinese menopausal women (1). The results showed significantly decreased STT-B completion time in the intervention group, indicating that the STT was sensitive to the therapeutic effect on mental flexibility. Recent studies used the STT to define objective cognitive impairment in processing speed and executive function in individuals with mild cognitive impairments (MCI) (2-4). In two studies, the MCI group performed worse than the normal cognition (NC) group in both STT-A and STT-B completion time, suggesting the ability of STT to capture cognitive profile differences between the two groups (3,4). Moreover, one of the studies (3) found that the STT-A completion time was significantly correlated with performance in the Category Fluency (CF) test, and the MCI group showed significantly impaired CF performance as compared to the NC group, both before and after controlling the STT-A time as a covariate. It suggested that the STT-A time was not a confounding factor for the impaired Category Fluency (CF) performance in MCI and the STT-A time provided a unique psychometric measurement of cognitive impairment in MCI (3).

Various research groups in China have adopted the STT to examine executive function in older adults (5–10) and patients with cognitive impairments (5–10), migraine (11), and sleep apnea (12). It was also adapted into a child version and assessed cognitive flexibility in Australian children (13). One study (10) provided the normative data of the STT for adults with NC, MCI, and Alzheimer’s disease (AD), showing that the STT completion time was accurate in distinguishing AD from NC (sensitivity = 76.4% – 92.4%, specificity = 66.4% – 75%). Several other studies examined the ability of the STT to identify individuals at risk of AD, such as those with MCI. MCI is a transitional stage between normal aging and dementia, affecting 16.0% – 18.9% of community-dwelling older adults (14,15). MCI individuals have a higher risk of progressing into dementia (annual conversion rate: 10–20%) than normal older adults (annual conversion rate: 1–2%) (16–18). Significant differences in STT completion times between the NC and MCI groups were consistently observed (5,8,19), and one study (20) using a machine learning approach achieved promising results in classifying AD from NC (sensitivity = 86.6%, specificity = 96.0%) and classifying MCI from NC (sensitivity = 68.0%, specificity = 75.0%).

Subjective memory impairment (SMI) refers to the subjective perception of memory or cognitive declines. It is prevalent in older adults, with 25%–50% of adults above 65 years and 88% of adults above 85 years reporting SMI (21). Although SMI individuals may perform within the normal range on neuropsychological assessments, they may still have subtle cognitive changes. SMI has been suggested as an important feature of preclinical AD and may precede the manifestation of MCI, therefore making it a potentially critical time window for early prediction and early intervention of AD (22–28). However, the association between SMI and AD or MCI is complex and under debate (29,30), and the existing body of substantive research investigating SMI was relatively limited in comparison to AD and MCI (24). Previous studies on the cognitive functions of SMI mainly focused on memory or general cognition (26), and the neuropsychological performance of SMI in executive function is still under-researched. As processing speed and executive function are affected early in MCI and AD (31), it is worth exploring whether the STT can also detect cognitive changes at the preclinical stage of AD.

While previous research on the STT provided information on the MCI and AD (5,8,10,19,20), the primary aim of the present study is to compare the STT performance between preclinical (i.e., SMI) and prodromal (i.e., MCI) stages of AD as well as compare these two stages against normal cognition. Therefore, the present study contributes to the literature by providing an understanding of the processing speed and executive function among the earliest stages at risk of AD. Another aim of the present study is to examine whether the combination of STT time and errors can better predict the groups as compared to individual STT measures. It was believed that early detections at the preclinical and prodromal stages of AD can contribute to the early prevention of progressing into AD (24,32). Also, relationships between the STT and demographics and other neuropsychological tests assessing different cognitive domains were examined.

## Method

### Participants

A total of 351 Chinese-speaking adults aged between 50 and 80 years were recruited through posting advertisements in community centers in Hong Kong and on social media. Those who had any of the following conditions were excluded: (1) a history of neurological disorders (including dementia, Parkinson’s disease, stroke, head injury, epilepsy and seizures, and brain tumor), psychiatric disorders, visual impairment, hearing impairment, or other physical disabilities that are severe enough to affect the test performance; (2) a prescription of psychiatric medications; (3) signs of dementia, defined as obtaining a score lower than 19 in the Hong Kong version of Montreal Cognitive Assessment (HK-MoCA) (33). After screening, 298 participants were eligible for the study.

According to the MCI criteria proposed by Bondi et al. (34), participants were classified into the MCI group if any one of the following criteria was met: (1) obtaining a score lower than 1 SD below the age-corrected normative mean on two measures within at least one cognitive domain including memory, language, or processing speed/executive function; (2) obtaining a score lower than 1 SD below the age-corrected normative mean in each of the three cognitive domains; (3) obtaining a score of 9 or above on the Functional Activity Questionnaire (FAQ) (35). Nevertheless, all the participants reported themselves as functionally independent in daily living activities, requiring minimal aid or assistance. 77 participants who met the MCI criteria were classified into the MCI group. The SMI group consisted of 145 participants who did not have significant objective cognitive impairments according to Bondi’s criteria of MCI (34) and demonstrated SMI, defined as obtaining a score of 3 or above in the Abbreviated Memory Inventory for Chinese (AMIC) (36). The remaining 76 participants without SMI and objective cognitive impairment were classified into the NC group. To match the age, education, and gender across three groups, a subsample of 70 participants was selected from each group, resulting in a total sample size of 210 participants for the subsequent data analysis.

### Measures and Procedures

Informed consent was obtained from the participants before the experiment. All participants underwent a battery of neuropsychological assessments and questionnaires. The HK-MoCA was administered to assess global cognition and screen participants with suspected dementia (20). The AMIC (36) was used to define subjective memory impairments. Neuropsychological assessments were performed to define MCI in three cognitive domains (i.e., memory, language, and speed/executive function). Memory domain was assessed using the Hong Kong List Learning Test (HKLLT) 10-min delayed recall, 30-min delayed recall, and recognition (37). Speed/executive function domain was evaluated using the STT completion time in STT-A and STT-B and the Five-Point Test (FPT) unique design (38,39). For the language domain, the number of unique words produced within 60-second in the animal and transportation condition of the Category Fluency (CF) (40) and the spontaneous naming in the 30-item Boston Naming Test (BNT) (41) were used. In addition, neuropsychological tests that assess attention and working memory (i.e., Digit Span, DS) (42) and visuospatial skills (i.e., Rey-Osterrieth complex figure, Rey-O) (43) were also performed to examine their correlations with the STT. The STT-A requires participants to connect numbers 1–25 in order, without considering the enclosing shape. The STT-B requires participants to connect numbers 1–25 in order, and meanwhile alternating between two different enclosing shapes. Details of the STT instructions and the practice trials were illustrated in supplementary materials (Figure S1). The STT measures included the completion time of the STT-A and STT-B, and the total number of errors committed in the STT-A and STT-B (i.e., errors in number, shape, prompt by the examiner if the participant could not find the next number within 10-second, and near miss errors).

### Data analysis

To compare the demographics (i.e., age, year of education, gender) and neuropsychological performances among the NC, SMI, and MCI groups, one-way ANOVA or the Chi-squared test was conducted depending on the nature of the variables (e.g., continuous or categorical). Post-hoc comparisons were performed to detect any significant between-group differences.

Furthermore, receiver operating characteristics (ROC) analysis was performed to evaluate the classification performance of each STT measure and the composite scores that combined different STT measures. The optimal cut-offs were selected based on Youden’s index (sensitivity + specificity – 1). To calculate the composite scores, stepwise binary logistic regression using the forward (likelihood ratio) method was employed to classify SMI from NC, MCI from NC, and MCI from participants without objective cognitive impairments (NC and SMI). The potential predictors included demographic variables (age, education, and gender) and STT measures (STT-A completion time, STT-B completion time, STT-A errors, and STT-B errors). Considering the potential tradeoff between the completion time and errors, interactions between completion time and errors (STT-A completion timexSTT-A errors, STT-B completion timexSTT-B errors) were also entered as potential predictors, resulting in a total of 9 potential predictors for the regression analysis. Additionally, the ability of STT to predict MCI individuals with impairment in memory and/or language domain against the NC/SMI group was also analyzed using stepwise regression analysis and ROC analysis.

To understand the relationship between demographics and STT performance within each group and the whole sample, Pearson’s correlation was calculated between the STT measures and age and education. The independent t-test was performed to examine any significant gender differences. To obtain demographic-adjusted STT performances, stepwise linear regression was performed using STT measures as dependent variables and age, education, and gender as predictors based on the NC group. In addition, Pearson’s correlation was performed to examine the relationship between STT and other neuropsychological tests assessing different cognitive domains (i.e., executive function, memory, working memory, language, attention and working memory, and visuospatial skills) within each group and the whole sample. Correlations between all the neuropsychological tests and demographics were examined to account for the potential confounding effects of demographics (i.e., age, education). Partial correlations between the STT measures and other neuropsychological tests were performed when demographics had a significant confounding effect. The false discovery rate (FDR) method was applied to adjust for the p-values to reduce the potential inflation of the type I error due to multiple comparisons.

## Results

### Demographics

As shown in Table 1, no significant difference was found among the three groups in terms of age (p = 0.75), year of education (p = 0.71), and gender (p = 0.78).

**Table 1:** Demographics and neuropsychological performance of the normal cognition (NC), subjective memory impairments (SMI), and mild cognitive impairments (MCI) groups

**Variables**	**NC (n = 70)**	**SMI (n = 70)**	**MCI (n = 70)**	F/X^2^	**df**	**p**	**η**^2^/**V**	**post-hoc**
Demographics								
	65.45 (6.29)	66.14 (6.49)	66.14 (5.90)	0.29	2, 207	0.75	0.003	
Education	12.5 (3.85)	12.47 (3.22)	12.04 (3.78)	0.35	2, 207	0.71	0.003	
Gender	44/26	48/22	46/24	0.51	2	0.78	0.049	
Neuropsychological performances								
*Memory*								
HKLLT 10min DR	10.6 (2.64)	9.83 (2.99)	7.24 (3.42)	23.51	2, 207	&lt; 0.001***	0.185	NC, SMI > MCI***
HKLLT 30min DR	10.47 (2.66)	9.63 (2.87)	7.29 (3.42)	21.16	2, 207	&lt; 0.001***	0.170	NC, SMI > MCI***
HKLLT recognition	86.16 (12.85)	87.91 (11.89)	81.25 (17.68)	2.80	2, 159	0.06	0.034	
*Executive function*								
STT-A time	46.64 (15.24)	47.29 (12.54)	56.25 (18.81)	8.16	2, 207	&lt; 0.001***	0.073	NC, SMI &lt; MCI***
STT-B time	113.96 (33.29)	116.09 (32.3)	131.69 (49.33)	4.29	2, 207	0.02*	0.040	NC, SMI &lt; MCI*
STT-A errors	0.16 (0.44)	0.14 (0.39)	0.04 (0.27)	1.96	2, 207	0.14	0.019	
STT-B errors	2.21 (4.3)	1.36 (1.54)	6.29 (7.11)	20.39	2, 207	&lt; 0.001***	0.165	NC, SMI &lt; MCI***
FPT unique design	24.83 (9.75)	26.61 (7.48)	22.17 (7.82)	3.10	2, 158	0.048*	0.038	SMI > MCI*
*Language*								
BNT	25.12 (2.72)	23.94 (2.94)	21.41 (4.4)	14.08	2, 154	&lt; 0.001***	0.155	NC, SMI > MCI***
CF-animal	19.13 (5.48)	18.76 (5.25)	16.46 (4.71)	5.51	2, 207	0.005**	0.051	NC**, SMI* > MCI
CF-transportation	13.83 (3.82)	13.01 (3.34)	11.8 (2.86)	6.45	2, 207	0.002**	0.059	NC**, SMI† > MCI
*Other cognitive domains*								
DS-forward span	7.87 (1.17)	8.00 (1.02)	7.87 (1.17)	0.22	2, 160	0.80	0.003	
DS-backward span	5.36 (1.55)	5.60 (1.72)	5.28 (1.52)	0.51	2, 160	0.60	0.006	
Rey-O copy	31.13 (3.42)	30.22 (5.22)	30.26 (6.15)	0.72	2, 207	0.49	0.007	
Rey-O IR	15.13 (6.46)	15.33 (5.18)	14.43 (5.63)	0.30	2, 207	0.75	0.004	
Rey-O DR	14.55 (6.21)	14.93 (5.09)	13.96 (5.26)	0.35	2, 207	0.71	0.004	

Note. BNT, Boston Naming Test; DR, delayed recall; DS, Digit Span; FPT, Five-point Test; HKLLT, Hong Kong List Learning; IR, immediate recall; Rey-O, Rey-Osterrieth Complex Figure; STT-A, Shape Trail Test – Part A; STT-B, Shape Trail Test – Part B. Numbers outside and within the brackets are mean and SD, respectively. † p &lt; 0.06, * p &lt; 0.05, ** p &lt; 0.01, *** p &lt; 0.001.

### STT performance of the NC, SMI, and the MCI groups

**Figure 1:**
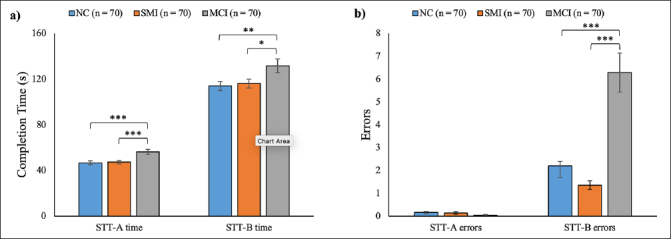
Performance in the Shape Trail Test (STT) of the normal cognition (NC), subjective memory impairments (SMI), and mild cognitive impairment (MCI) groups. a) Completion time of STT-A and STT-B. b) Number of errors committed in the STT-A and STT-B Note. * p &lt; 0.05, ** p &lt; 0.01, *** p &lt; 0.001. The error bar represents 1 SE ± mean.

As shown in Figure 1a, the completion time of STT-A (p < 0.001) and STT-B (p = 0.015) were significantly different among the three groups. Post-hoc analyses indicated that there was no significant difference between the NC (M = 46.64, SD = 15.24) and SMI (M = 47.29, SD = 12.54) groups in the STT-A completion time (p = 0.81), nor in the STT-B completion time (NC: M = 113.96, SD = 33.39; SMI: M = 116.09, SD = 32.30; p = 0.75). However, both the NC and SMI groups showed significantly shorter completion times than the MCI group (STT-A: M = 56.25, SD = 18.81; STT-B: M = 131.69, SD = 49.33) (STT-A: NC vs MCI: p < 0.001, SMI vs MCI: p < 0.001; STT-B: NC vs MCI: p = 0.008, SMI vs MCI: p = 0.019).

Regarding the errors (Figure 1b), no significant group difference was found in the number of errors committed in STT-A (p = 0.14) among NC (M = 0.16, SD = 0.44), SMI (M = 0.14, SD = 0.39), and MCI (M = 0.04, SD = 0.27). However, significant group differences were observed in the STT-B errors (p < 0.001). Post-hoc analyses revealed a similar number of errors committed in STT-B by the NC (M = 2.21, SD = 4.30) and SMI (M = 1.36, SD = 1.54) groups (p = 0.30). Both the NC (p < 0.001) and SMI (p < 0.001) groups committed significantly fewer errors in STT-B than the MCI group (M = 6.29, SD = 7.11).

### Classification accuracy of the NC, SMI, and MCI groups

**Figure 2:**
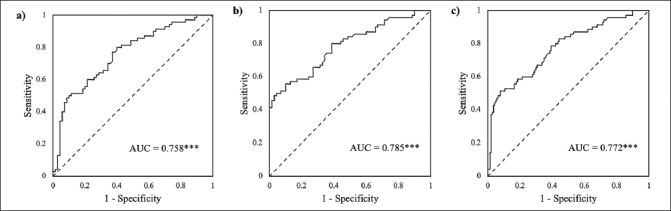
Receiver operating characteristics (ROC) curves of the composite scores combining STT-B completion time, STT-A errors, and STT-B completion timexSTT-B errors for classifying the mild cognitive impairment (MCI) group from the normal cognition (NC) and subjective memory impairments (SMI) groups. a) MCI vs NC. b) MCI vs SMI. c) MCI vs NC + SMI Note. AUC = area under the curve. *** p &lt; 0.001.

Table 2 and Figure 2 presented the performance of different STT measures and their composite scores in classifying the NC, SMI, and MCI groups.

**Table 2:** Classification results of the Shape Trail Test (STT) for the normal cognition (NC), subjective memory impairments (SMI), and mild cognitive impairment (MCI) groups

**Predictor**	**Cut-off**	**Sensitivity**	**Specificity**	**AUC**	p
NC/SMI					
STT-A time	47.95	48.6%	62.9%	0.539	0.43
STT-B time	95.88	77.1%	38.6%	0.519	0.70
STT-A errors	0.5	12.9%	87.1%	0.499	0.99
STT-B errors	0.5	62.9%	42.9%	0.511	0.82
NC/MCI					
STT-A time	51.07	60.0%	68.6%	0.650	0.002**
STT-B time	92.99	84.3%	32.9%	0.590	0.048*
STT-A errors	3	0.0%	100.0%	0.451	0.31
STT-B errors	8	38.6%	92.9%	0.629	0.009**
Composite score	-0.42	80.0%	60.0%	0.758	&lt; 0.001***
SMI/MCI					
STT-A time	51.07	60.0%	65.7%	0.633	0.007***
STT-B time	124.13	45.7%	71.4%	0.579	0.11
STT-A errors	1.5	1.4%	98.6%	0.451	0.31
STT-B errors	7.5	38.6%	100.0%	0.633	0.007**
Composite score	-0.54	80.0%	61.4%	0.785	&lt; 0.001***
NC+SMI/MCI					
STT-A time	51.07	60.0%	67.1%	0.641	0.001**
STT-B time	104.65	70.0%	56.4%	0.584	0.046*
STT-A errors	3	0.0%	100.0%	0.451	0.24
STT-B errors	8	38.6%	96.4%	0.631	0.002**
Composite score	−1.13	78.6%	60.7%	0.772	&lt; 0.001***

Note. AUC = Area under the curve. * p &lt; 0.05, ** p &lt; 0.01, *** p &lt; 0.001.

#### Classifying the SMI from the NC group

None of the four STT measures demonstrated significant discrimination ability between the SMI and NC groups (AUC = 0.499 – 0.539, p = 0.43 – 0.99). Stepwise regression analysis also indicated that neither the individual STT measures nor their interactions (p = 0.11 – 0.92) significantly predicted the classification of the SMI and NC groups.

#### Classifying the MCI from the NC group

When using individual STT measures to classify the MCI from the NC group, the STT-A completion time outperformed other STT measures, showing a significant moderate discrimination ability (AUC = 0.650, p = 0.002). At the cut-off of 51.07, a sensitivity of 60.0% and specificity of 68.8% was obtained. The STT-B completion time (AUC = 0.590, p = 0.048) and STT-B errors (AUC = 0.629, p = 0.009) also showed statistically significant discrimination ability. However, the optimal cutoffs for these two STT measures showed either low sensitivity or low specificity (STT-B time: sensitivity = 84.3%, specificity = 32.9%; STT-B errors: sensitivity = 38.6%, specificity = 92.9%). The STT-A errors did not differentiate between the MCI and NC groups (p = 0.31). Stepwise regression revealed that the STT-B completion time (p = 0.004), the STT-A errors (p = 0.022), and the interaction between STT-B completion time and STT-B errors (p < 0.001) were significant predictors for classifying the MCI and NC groups. In contrast, demographics, other STT measures, and the interaction between STT-A completion time and STT-A errors were not significant predictors (p = 0.16 – 0.83). The equation of composite score derived from three significant predictors was provided below:

Composite score (NC vs MCI) = −2.102 + 0.013 · STT-B time − 1.550 × STT-A errors + 0.001 × STT-B time × STT-B errors

As shown in Figure 2a, this composite score exhibited good discrimination between the MCI and NC groups (AUC = 0.758, p < 0.001), and provided a sensitivity of 80.0% and a specificity of 60.0% at the cut-off of −0.42.

#### Classifying the MCI from the SMI group

Similar to the classification between MCI and NC, the STT-A completion time was the most discriminative individual STT measure (AUC = 0.633, p = 0.007). It achieved a sensitivity of 60.0% and a specificity of 65.7% at the cut-off of 51.07. The STT-B errors also showed moderate discrimination ability (AUC = 0.633, p = 0.007). Nevertheless, the optimal cut-off of 7.5 for STT-B errors yielded low sensitivity (38.6%) but high specificity (100.0%). The STT-B completion time (p = 0.11) and STT-A errors (p = 0.31) were not significant factors for the classification. Regarding the composite score, stepwise regression also suggested three significant predictors including the STT-B completion time (p = 0.003), the STT-A errors (p = 0.010), and the interaction between STT-B completion time and STT-B errors (p < 0.001), while other predictors were not significant (p = 0.24 – 0.96). The equation is provided below:

Composite score (SMC vs MCI) = −2.489 + 0.015 × STT-B time − 2.074 × STT-A errors + 0.002 × STT-B time × STT-B errors

As shown in Figure 2b, this composite score demonstrated good discrimination ability (AUC = 0.785, p < 0.001). At the cut-off of −0.54, the composite score achieved a sensitivity of 80.0% and a specificity of 61.4%.

### Classifying the MCI from the combined NC and SMI group

Similar results were yielded when classifying participants with MCI from the combined NC and SMI group. Again, the most discriminative individual measure was STT-A time (AUC = 0.641, p = 0.001), which outperformed the STT-B errors (AUC = 0.631, p = 0.002), STT-B completion time (AUC = 0.584, p = 0.046), and STT-A errors (p = 0.24). Stepwise regression analysis suggested that a combination of the STT-B completion time (p = 0.001), the STT-A errors (p = 0.010), and the interaction between STT-B completion time and STT-B errors (p < 0.001) was more predictive of the group membership than using single STT measures. All the other predictors were not significant (p = 0.11 – 0.91). The equation is provided below:

Composite score (NC+SMC vs MCI) = −2.918 + 0.013 × STT-B time − 1.663 × STT-A errors + 0.002 × STT-B time × STT-B errors

This composite score also showed a good discrimination ability with an AUC of 0.007 (p < 0.001) (Figure 2c). At the cut-off of −1.13, the composite score obtained a sensitivity of 78.6% and a specificity of 60.7%.

#### Classifying the MCI with memory and/or language impairment from the NC/SMI group

To further explore the performance of STT in classifying MCI individuals with impairment in cognitive domains other than executive function, those with impairment in memory and/or language domain were extracted from the MCI group and discriminated against the NC/SMI group. 76% of the participants (53 out of 70) in the MCI group showed cognitive impairment in memory and/or language. To match the group size, a subsample of 53 participants was randomly selected from the NC and SMI groups, respectively. When discriminating MCI individuals with memory and/or language impairment against the NC group, stepwise regression analysis suggested a composite score of STT-B completion time (p = 0.007) and STT-B errors (p = 0.032). ROC analysis on this composite score demonstrated a sensitivity of 67.9% and a specificity of 60.4% at the optimal cut-off of 1.28, and an AUC of 0.69 (p = 0.001). When discriminating against the SMI group, stepwise regression analysis suggested a composite score of STT-A errors (p = 0.02), STT-B completion time (p = 0.03), and STT-B completion timexSTT-B errors interaction (p = 0.002). This composite score achieved a sensitivity of 75.5% and a specificity of 58.5% at the optimal cut-off of −0.31, and an AUC of 0.72 (p < 0.001). The equations for calculating the composite scores are provided below:

Composite score (NC vs MCI_memory and/or language impairment_) = −2.031 + 0.014 × STT-B time + 0.083 × STT-B errors

Composite score (SMC vs MCI_memory and/or language impairment_) = −1.799 – 1.801 × STT-A errors + 0.011 × STT-B time + 0.002 × STT-B time × STT-B errors

### Relationship between STT and demographics

As shown in Table 3, age was significantly correlated with the completion time of both STT-A and STT-B within the whole sample (p < 0.001) and within each group (p < 0.05). Similarly, education was significantly correlated with the completion time in both STT-A and STT-B within the whole sample (STT-A: p < 0.001; STT-B: p < 0.01) and within each group (p < 0.05). In contrast, gender difference was not significant in the completion time within the whole sample (STT-A: p = 0.36, STT-B: p = 0.20) and within each group (p = 0.08 – 0.94).

**Table 3:** Correlations between the Shape Trail Test (STT) and demographics and other neuropsychological tests in the normal cognition (NC), subjective memory impairment (SMI), and mild cognitive impairment (MCI) groups and the whole sample

**Variable**	**NC**		**SMI**		**MCI**		**Whole sample**	
	**[1]**	**[2]**	**[1]**	**[2]**	**[1]**	**[2]**	**[1]**	**[2]**
[1] STT-A time	-		-		-		-	
[2] STT-B time	0.62*** [0.33, 0.68]	-	0.64*** [0.30, 0.67]	-	0.62*** [0.55, 0.81]	-	0.64*** [0.44, 0.67]	-
Demographics								
[3] Age	0.29* [−0.09, 0.49]	0.26* [0.02, 0.48]	0.54*** [0.21, 0.67]	0.37** [0.20, 0.59]	0.46*** [0.24, 0.63]	0.48*** [0.23, 0.64]	_0.41***_ [0.17, 0.51]	0.37*** [0.24, 0.47]
[4] Education	0.38** [−0.54, −0.18]	−0.31* [−0.51, −0.06]	−0.44** [−0.59, −0.13]	−0.36** [−0.45, 0.01]	−0.27* [−0.49, −0.03]	−0.40** [−0.60, −0.16]	−0.35*** [−0.47, −0.22]	−0.36*** [−0.49, −0.23]
Executive function								
[5] FPT	−0.41* [−0.54, −0.15]	−0.39* [−0.54, −0.11]	−0.33 [−0.59, −0.01]	−0.41* [−0.63, −0.14]	−0.48* [−0.65, −0.26]	−0.31 [−0.50, −0.10]	−0.40*** [−0.51, −0.26]	−0.33*** [−0.45, −0.19]
Memory								
[6] HKLLT 10min delayed recall	−0.13 [−0.31, 0.06]	−0.29 [−0.47, −0.06]	−0.38* [−0.44, −0.03]	−0.27 [−0.40, 0.13]	0.28 [0.15, 0.63]	0.09 [–0.14, 0.42]	−0.12 [−0.19, 0.11]	−0.18* [−0.29, 0.03]
[7] HKLLT 30min delayed recall	−0.23 [−0.42, −0.05]	−0.32+ [−0.48, −0.10]	−0.34* [−0.44, 0.05]	−0.31* [−0.47, 0.09]	0.20 [0.19, 0.55]	0.06 [−0.03, 0.46]	−0.08 [−0.19, 0.12]	−0.12 [−0.27, 0.04]
Language								
[8] BNT	−0.16 [−0.39, 0.14]	−0.31 [−0.60, 0.08]	−0.38* [−0.58, −0.14]	−0.35* [−0.53, −0.14]	−0.39* [−0.57, 0.13]	−0.39+ [−0.63, 0.22]	−0.41*** [−0.55, −0.25]	−0.45*** [−0.59, −0.30]
[9] CF - animal	−0.28 [−0.47, −0.10]	−0.26 [−0.48, −0.02]	−0.15 [−0.35, 0.08]	−0.19 [−0.37, 0.03]	−0.20 [−0.48, 0.17]	−0.10 [−0.49, 0.16]	−0.18* [−0.32, −0.05]	−0.17* [−0.32, −0.01]
[10] CF - transportation	−0.35* [−0.55, 0.02]	−0.27 [−0.44, 0.04]	−0.23 [−0.42, 0.00]	−0.27 [−0.45, −0.08]	−0.16 [−0.43, 0.26]	−0.14 [−0.36, 0.22]	−0.26*** [−0.38, −0.07]	−0.24** [−0.32, −0.02]
Attention & working memory								
[11] DS-Forward	−0.11 [−0.32, 0.11]	−0.22 [−0.43, 0.00]	−0.32 [−0.59, 0.04]	−0.15 [−0.42, 0.13]	−0.10 [−0.38, 0.18]	−0.09 [−48, 0.24]	−0.13 [−0.28, 0.01]	−0.15 [−0.31, 0.01]
[12] DS-Backward	−0.24 [−0.39, 0.03]	−0.39* [−0.51, −0.15]	−0.22 [−0.48, 0.06]	−0.38+ [−0.60, −0.12]	−0.03 [−0.29, 0.30]	0.01 [−0.27, 0.39]	−0.12 [−0.26, 0.02]	−0.24** [−0.37, −0.10]
Visuospatial skills								
[13] Rey-O copy	−0.10 [−0.17, 0.19]	−0.14 [−0.26, 0.16]	−0.03 [−0.20, 0.14]	−0.05 [−0.21, 0.10]	−0.28 [−0.43, 0.06]	−0.13 [−0.24, 0.24]	−0.08 [−0.31, 0.11]	−0.02 [−0.32, 0.13]
[14] Rey-O immediate recall	−0.27 [−0.39, 0.02]	−0.35* [−0.51, −0.03]	−0.30 [−0.54, 0.05]	−0.30 [−0.48, −0.08]	0.06 [−0.59, 0.55]	−0.04 [−0.73, 0.42]	−0.11 [−0.41, −0.01]	−0.22* [−0.50, −0.19]
[15] Rey-O delayed recall	−0.30+ [−0.51, −0.07]	−0.32+ [−0.51, −0.10]	−0.15 [−0.45, 0.18]	−0.15 [−0.41, 0.12]	0.07 [−0.29, 0.42]	−0.08 [−0.38, 0.31]	−0.12 [−0.39, 0.04]	−0.19* [−0.47, −0.14]

Note. BNT, Boston Naming Test; CF, Category Fluency; DS, Digit Span; FPT, Five-point Test; HKLLT, Hong Kong List Learning Test; Rey-O, Rey-Osterrieth Complex Figure. Numbers in brackets represent 95% bootstrapped confidence interval. + p &lt; 0.06, * p &lt; 0.05, ** p &lt; 0.01, *** p &lt; 0.001.

Besides, stepwise linear regression was performed to calculate demographic-adjusted STT performance based on the NC group (see supplementary material Table S1). The equations were shown below:

STT-A completion time: Adjusted score = raw score − 0.696 × age + 1.517 × education

STT-B completion time: Adjusted score = raw score – 1.351 × age + 2.650 × education

### Correlations between STT and other neuropsychological tests

Table 3 showed the partial correlation between STT and other neuropsychological tests within each group and the whole sample, after controlling the potential confounding effect of age and/or education. The results were organized by different cognitive domains:

#### Executive function

The FPT unique design was significantly correlated with STT-A completion time (p = 0.020) and STT-B completion time (p = 0.024) in the NC group, with a moderate to large effect size. The FPT unique design was only significantly correlated with STT-B completion time (p = 0.033) in the SMI group and with STT-A completion time (p = 0.022) in the MCI group. As a whole sample, the FPT also demonstrated significant moderate correlations with the STT completion times (p < 0.001).

#### Memory

The HKLLT 10min delayed recall only showed a significant moderate correlation with STT-A completion time in the SMI group (p = 0.021). The HKLLT 30-minute delayed recall was marginally significantly correlated with STT-B completion time (p = 0.052) in the NC group, and significantly correlated with STT-A completion time (p = 0.034) and STT-B completion time (p = 0.048) with moderate effect sizes in the SMI group. Within the whole sample, only STT-B showed a significant but weak correlation with HKLLT 10min delayed recall (p = 0.02).

#### Language

The BNT spontaneous naming showed significant correlations with STT-A completion time in the SMC (p = 0.019) and MCI (p = 0.048) groups, as well as significant or marginal significant correlation with STT-B completion time in the SMC (p = 0.031) and MCI (p = 0.050) groups, with moderate effect sizes. Regarding the CF test, only a significant moderate correlation between CF transportation condition and STT-A completion time was observed in the NC group (p = 0.033). Within the whole sample, the CF showed significant but weak correlations with the STT completion times (p < 0.05).

#### Attention and working memory

The DS backward (assessing working memory) was significantly correlated with the STT-B completion time (p = 0.020) with a moderate effect size in the NC group. Similar results were found in the SMI group, as the DS backward showed a marginally significant moderate correlation with STT-B completion time (p = 0.051). Within the whole sample, only a significant but weak correlation was observed between the STT-B completion time and DS backward.

#### Visuospatial skills

In the NC group, the Rey-O immediate recall showed a significant moderate correlation with STT-B completion time (p = 0.031), and the Rey-O delayed recall showed marginally significant moderate correlations with the completion time in STT-A (p = 0.058) and STT-B (p = 0.051). Among all the participants, only the STT-B completion time demonstrated significant but weak correlations with Rey-O immediate recall (p = 0.010) and delayed recall (p = 0.022).

## Discussion

The present study provided insights into the STT performance in individuals with NC, SMI, and MCI. The NC and SMI groups demonstrated similar performance on all STT measures, while the MCI group showed significantly poorer performance in the completion time of STT-A and STT-B and STT-B errors, compared to both the NC and SMI groups. Besides, combining different STT measures showed good discrimination between the MCI group and the other two groups (AUC = 0.758 – 0.785, sensitivity = 78.6% – 80.0%, specificity = 60.0% – 61.4%). However, the STT was unable to classify between the SMI and NC groups.

To our best knowledge, this is the first study to examine the STT performance in SMI. Although the SMI showed a slightly longer completion time in both STT-A and STT-B than the NC group, it did not achieve statistical significance. It is possible that at the early stage of SMI, cognitive changes related to processing speed and mental flexibility may not yet manifest to be captured by the STT. This finding aligned with a previous study reporting TMT-B as an insensitive index for classifying SMI from NC (24). Considering the limited body of literature on the neuropsychological performance in executive function among individuals with SMI, the present study contributes to the literature by providing some initial results about the STT performance in SMI and indicating that the processing speed and mental flexibility may remain intact at the preclinical stage of AD. Another potential reason why the STT failed to discriminate between the NC and SMI could be the test/ criteria used for defining the SMI group. The 5-item AMIC was used for defining the SMI group in the present study. Due to the limited range of the AMIC total score, it was difficult to assess the variability within the SMI group. In future studies, the original full version of the 27-item Memory Inventory for the Chinese (MIC) (44) may allow us to assess a more variable level of memory complaints in participants and to examine whether there is a correlation between STT performance and the level of subjective memory impairments on a continuous scale.

In differentiating between MCI and NC/SMI, completion times in both STT-A and STT-B were significant factors, which was consistent with previous studies (3–5,8,10,19,20). In addition, STT-B error was also a significant factor, with the MCI group committing more errors in the STT-B than the NC and SMI groups. This highlights the importance of considering both completion time and errors in the STT, expanding on previous studies that primarily focused on the completion time. Moreover, our finding suggested that the STT could significantly differentiate between cognitively unimpaired (i.e., NC and SMI) and cognitively impaired (i.e., MCI), regardless of the subjective awareness of memory problems.

The present study assessed the performance of individual STT measures and their combination for classifying the NC, SMI, and MCI groups. STT-A completion time consistently showed moderate discriminatory power for classifying the MCI group from the other two groups (sensitivities, specificities > 60%), while other individual STT measures did not demonstrate sufficient discriminatory ability for the MCI group. Composite scores combining STT-B completion time, STT-A errors, and the interaction between STT-B completion time and STT-B errors were identified as the most significant indices for differentiating the MCI group from the other two groups. It yielded moderate discriminatory power, with AUCs ranging from 0.76 to 0.79 (p < 0.001), sensitivities around 80%, and specificities around 60%. However, the STT could not significantly differentiate the SMI group from the NC group, regardless of the specific STT measures and their interactions used.

Regarding the relationship between STT performance and demographic variables, age and education were found to be significant factors correlated with the completion time in STT-A and STT-B across three groups, which was consistent with a previous study by Zhao et al. (10). To facilitate future clinical utilization, the present study provided age- and education-adjusted STT scores based on the NC group, allowing for comparisons of STT performance across different ages and educational levels.

Moreover, the construct validity of the STT was demonstrated by examining the partial correlations between STT and other neuropsychological tests after controlling age and education. The relationships between STT and other neuropsychological tests varied across cognitive domains and cognitive stages (i.e., NC, SMI, MCI). Executive function domain (i.e., FPT unique design) showed the most consistent correlations with STT measures, particularly in the NC group, with moderate to large effect sizes. Correlations with other cognitive domains were also observed. For example, STT-B completion time was significantly or marginally significantly correlated with memory (HKLLT 30min delayed recall) and working memory (DS backward) measures in the NC and SMI groups, with moderate effect sizes. In contrast, the STT-A completion time showed limited correlations with memory and working memory. This suggests that the number-shape alteration involved in the STT-B may engage more memory and working memory processes compared to STT-A, leading to a stronger association between STT-B and memory and working memory measures. Overall, correlations with cognitive domains other than executive function were generally weak to moderate, suggesting a limited association between the STT and these cognitive domains. Since executive function is one of the sensitive indexes for detecting MCI and dementia (45), the correlation between STT and another executive function measure (i.e., FPT) further supported the clinical utilization of STT in classifying MCI. Further research is needed to understand the underlying cognitive processes involved in STT-A and STT-B and their unique contributions to different cognitive domains.

While the number of STT-B errors was significantly different between the NC and MCI groups, there was no significant group difference in the number of STT-A errors. It is worth noticing that the interpretation of STT errors in isolation should be approached with caution. Previous research on a similar test (TMT) suggested that it may be difficult to interpret errors in isolation, as errors were also commonly committed by normal controls (46). The completion time may partially account for errors, as additional time was required when errors were identified and corrected by the examiner during the test. Therefore, previous studies on the TMT or STT mainly examined the completion time instead of errors. In the present study, combining STT completion time and errors improved the classification accuracy for the MCI group compared to using STT measures alone. The stepwise regression analysis indicated that the interaction between the STT-B completion time and STT-B errors provided additional value for group classification. A longer STT-B completion time with more STT-B errors was associated with a higher risk of MCI, while more STT-A errors were associated with a lower probability of MCI. One potential explanation is that the STT-A errors may be influenced by a trade-off between completion time and accuracy, and it may be more common in normal adults. In contrast, the STT-B errors may reflect cognitive abilities more directly, as the STT-B imposes higher demands on mental flexibility. Future research may explore the combination of STT completion time and errors for better prediction of MCI.

Several clinical implications of the present study should be highlighted. Firstly, unlike a previous study which only reported the classification accuracy for MCI (20), the present study provided equations of composite scores and cut-offs for predicting MCI, enabling future application of STT in the clinical setting as a fast-screening test for MCI. There was evidence showing that early interventions at the stage of MCI could be more effective than interventions at the stage of AD (47). Therefore, the STT can contribute to the early detection of prodromal AD, which in turn allows the early intervention at the prodromal stage, slows down or prevents the progression to AD, and promotes quality of life. In addition, the present study provided demographic-adjusted STT for future use in different age and educational groups and established a norm for Chinese-speaking older adults in Hong Kong. Besides, the cultural fairness of the STT enables its utilization across diverse populations.

The present study has several limitations. First, the MCI group was diagnosed based on the performance on a set of neuropsychological tests, and STT was used as one of the tests for defining impairment in the executive function domain. Therefore, a tautological problem was introduced when analyzing the ability of STT to predict the MCI diagnosis, resulting in a potential overestimation in the classification accuracy. To disentangle the true classification accuracy from the potentially overestimated accuracy, the present study examined the ability of STT to predict MCI subtypes with impairments in memory and/or language domain and found a lower discriminatory power (AUC = 0.69, 0.72) as compared to the discriminatory power for predicting all-type MCI (AUC = 0.76 – 0.79). Future studies are needed to recruit an MCI sample that is diagnosed based on tests other than the STT and rigorous clinical procedures, as well as to examine the ability of STT for different MCI subtypes with a larger group size. Another limitation of the present study is the paper-and-pencil version of STT. Existing literature has shown that digital versions of TMT were sensitive for detecting MCI and dementia (45). Therefore, future studies could explore the validity and discrimination ability of a digital version of the STT for SMI, MCI, and dementia, considering the advantages of digital tests such as broader accessibility, faster screening for cognitive impairments, and incorporation of more digital features. It is worth noticing that in the present study, the discriminant power of the STT for predicting MCI, as measured by AUC, was only moderate in magnitude. Besides, the specificity was only around 60%. Future studies are warranted to examine whether the discriminant power and specificity can be improved when traditional STT measures are combined with digital features collected in the digital version of STT.

To summarize, the present study demonstrated that the STT is a validated, culture-neutral test for measuring processing speed and mental flexibility among older adults with NC, SMI, and MCI. The STT test was sensitive for differentiating the prodromal stage of AD (i.e., MCI) from NC or SMI, but insensitive for capturing cognitive change at the preclinical stage of AD (i.e., SMI). The importance of combining STT completion time and errors for better classification in the future was highlighted. In addition, the consistent correlations between the STT and executive function demonstrated the construct validity of STT and provided valuable information for future cognitive screening considering that executive function deficits are commonly associated with various neurological and psychiatric conditions.

### Supplementary Materials

Appendix

Appendix

## Data Availability

*Data Sharing:* The data that support the findings of this study will be available from the corresponding author upon reasonable request.

## Notes

*Ethics:* The study was performed in accordance with the Declaration of Helsinki and has been approved by the Joint Chinese University of Hong Kong–New Territories East Cluster Clinical Research Ethics Committee (CREC Ref. No.: 2022.633).
