# Supplementary material for: The Shape Trail Test Is Sensitive in Differentiating Older Adults with Mild Cognitive Impairment: A Culture-neutral Five-minute Test
Source: J Prev Alzheimers Dis. 2024 May 2;11(4):1166–76. doi: 10.14283/jpad.2024.80 (PMC11266266; doi:10.14283/jpad.2024.80)
Supplement: Supplementary file 1 — Appendix [file mmc1.docx]

**Table S1.** **Stepwise linear regression models for adjusting age, education, and gender for Shape Trail Test (STT) performance in the normal cognition (NC) group.**

| STT measure | Predictor |  | Coefficients | | | |  | Model summary | | | |
| --- | --- | --- | --- | --- | --- | --- | --- | --- | --- | --- | --- |
|  |  |  | *B* | *t* | *p* | *95% CI* |  | $R^{2}$ | *F* | *df* | *p* |
| STT-A time | (Constant) |  | 20.042 | 1.12 | 0.27 | -15.66 – 55.74 |  | 0.23 | 9.96 | 2, 67 | < 0.001 |
|  | Age |  | 0.696 | 2.68 | 0.009 | 0.18 – 1.22 |  |  |  |  |  |
|  | Education |  | -1.517 | -3.57 | < 0.001 | -2.37 – -0.67 |  |  |  |  |  |
| STT-B time | (Constant) |  | 58.679 | 1.44 | 0.16 | -22.78 – 140.14 |  | 0.16 | 6.33 | 2, 67 | 0.003 |
|  | Age |  | 1.351 | 2.28 | 0.026 | 0.17 – 2.53 |  |  |  |  |  |
|  | Education |  | -2.650 | -2.73 | 0.008 | -4.59 – -0.72 |  |  |  |  |  |

*Note*. CI, confidence interval.
