# Supplementary material for: The Shape Trail Test Is Sensitive in Differentiating Older Adults with Mild Cognitive Impairment: A Culture-neutral Five-minute Test
Source: J Prev Alzheimers Dis. 2024 May 2;11(4):1166–76. doi: 10.14283/jpad.2024.80 (PMC11266266; doi:10.14283/jpad.2024.80)
Supplement: Supplementary file 2 — Appendix [file mmc2.docx]

**Supplementary Materials**

**Figure Legends**

**Figure S1. Illustration of the Shape Trail Test.**

**a) Shape Trail Test – Part A (STT-A) practice trial.**

Instruction: “Here are some numbers (the examiner points to the example below the box). Please use the pen to connect the numbers in ascending order, from 1 to 2, then to 3, and until 8 (the examiner connects the numbers in the example as a demonstration). Please connect the numbers as fast and as accurate as possible with one stroke, and do not lift the pen tip from the paper. The line you draw needs to pass through the numbers. You will be reminded when making an error, and asked to go back to the previous number and connect to the correct number. Now please put the pen tip on the number 1 (the examiner points to the number 1 in the box) and start.”

**b) Shape Trail Test – Part B (STT-B) practice trial.**

Instruction: “Please also connect the numbers from 1 to 8 in ascending order (The examiner points to the example below the box). Different from the one you performed before, each number are enclosed by two different shapes including square and circle. Please start from the number 1 enclosed in square, and connect to the number 2 enclosed in circle, and then connect to the number 3 enclosed in square, and so on, alternating between squares and circles (the examiner connect the numbers in the example as a demonstration). Please perform as fast and as accurate as possible with one stroke, and do not lift the pen tip from the paper. The line you draw needs to pass through the numbers. You will be reminded when making an error, and asked to go back to the previous number and connect to the correct number. Now please put the pen tip on the number 1 (the examiner points to the number 1 in the box) and start.”

**Figure S1.**

**a) b)**

**
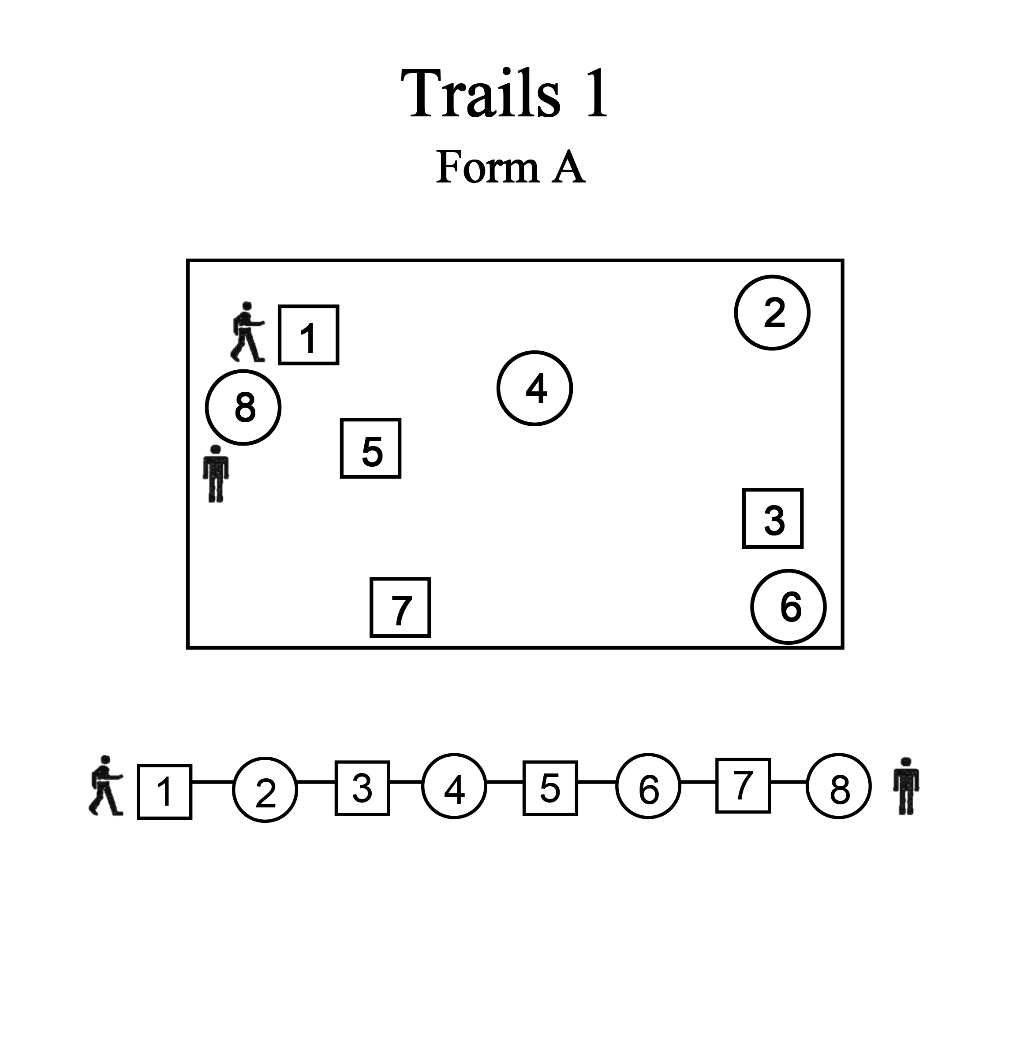

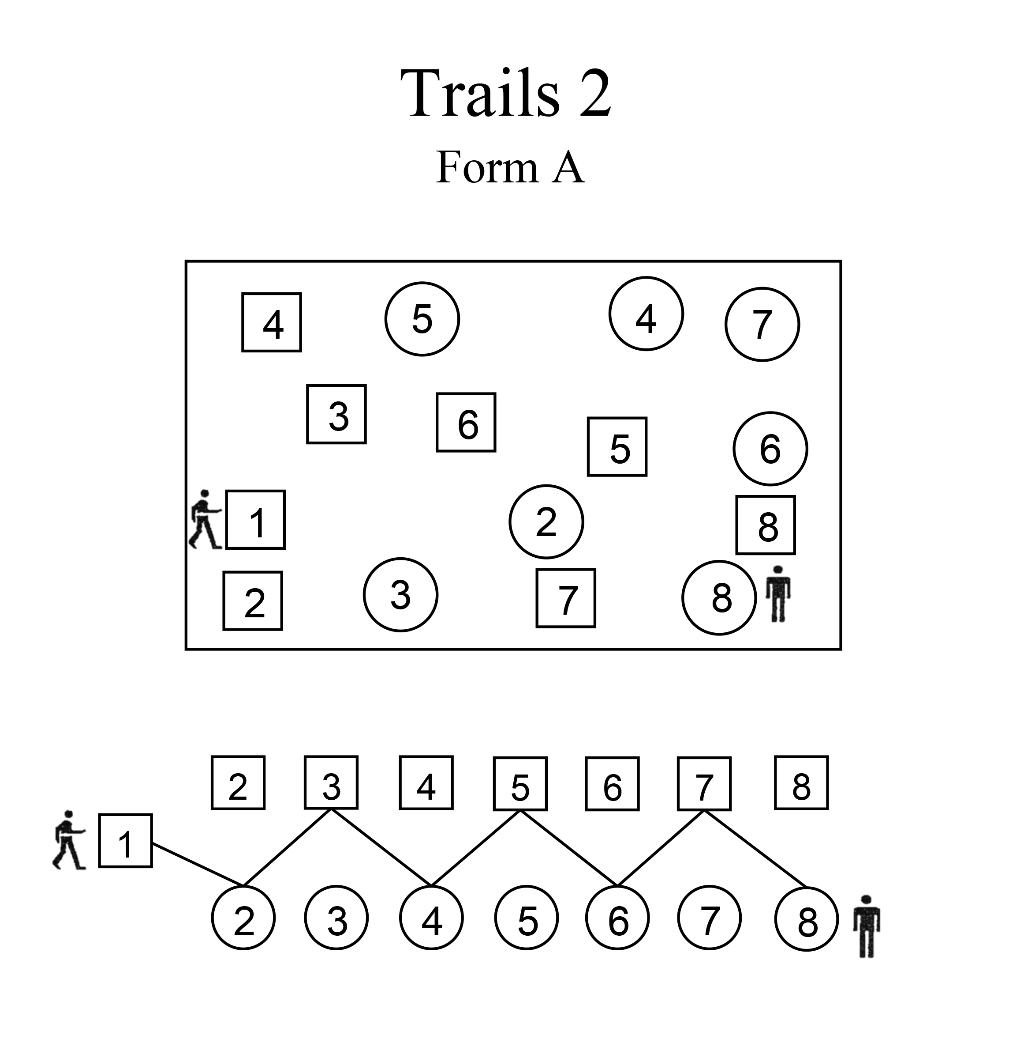
**
